# Targeting Oncogenic KRAS Using Peptide Nucleic Acid Oligomers Attached to Cell-Penetrating Peptides

**DOI:** 10.3390/ijms27167158

**Published:** 2026-08-10

**Authors:** Jayati Mondal, Dennis Lam, Termika O. Alcindor, Mary E. Gerritsen, Tilmann M. Brotz, Jodi Kennedy, Bruce Rehlaender, Arthur J. Ross, Daniel E. Levy, Christopher A. Bonagura, William N. Lanzilotta, Frank McCormick, Jeffrey H. Rothman, Andrew L. Wolfe

**Affiliations:** 1 Department of Biological Sciences, Hunter College, City University of New York, New York, NY 10065, USA; 2 Yalow Scholars Program, Hunter College, City University of New York, New York, NY 10065, USA; 3 Damon Runyon Cancer Research Foundation, Scholars Program for Advancing Research and Knowledge, New York, NY 10006, USA; 4 Maximizing Access to Research Careers Program, Hunter College, City University of New York, New York, NY 10065, USA; 5 McNulty Scholars Program, Hunter College, City University of New York, New York, NY 10065, USA; 6 Transfer to STEM Student Success Program, Hunter College, City University of New York, New York, NY 10065, USA; 7 Oncogenuity, Inc., New York, NY 10014, USA; 8 Gerritsen Consulting, San Mateo, CA 94402, USA; 9 PharmaDirections, Cary, NC 27513, USA; 10 Fortress Biotech, Waltham, MA 02453, USA; 11 DEL BioPharma, LLC, San Mateo, CA 94402, USA; 12 Art Robbins Instruments, Sunnyvale, CA 94089, USA; 13 Department of Chemistry, University of Georgia, Athens, GA 30602, USA; 14 Helen Diller Family Comprehensive Cancer Center, University of California San Francisco, San Francisco, CA 94158, USA; 15 National Cancer Institute RAS Initiative, Cancer Research Technology Program, Frederick National Laboratory for Cancer Research, Frederick, MD 21701, USA; 16 Department of Medicine, Icahn School of Medicine at Mount Sinai, New York, NY 10029, USA; 17 Herbert Irving Comprehensive Cancer Center, Department of Medicine, Columbia University School of Medicine, New York, NY 10032, USA; 18 Weill Cornell Medicine, Department of Pharmacology, New York, NY 10065, USA; 19 Molecular, Cellular, and Developmental Biology Ph.D. Subprogram, Graduate Center of the City University of New York, New York, NY 10016, USA; 20 Biochemistry Ph.D. Program, Graduate Center of the City University of New York, New York, NY 10016, USA

**Keywords:** peptide nucleic acid, PNA, cell-penetrating peptide, CPP, KRAS, mutant, G12D, lung cancer, pancreatic cancer, NSCLC, PDAC, inhibition, therapy

## Abstract

Approximately 30% of tumors contain an activating mutation in the oncogene KRAS, leading to increased cell proliferation that often promotes non-small cell lung cancers, colorectal adenocarcinomas, pancreatic ductal adenocarcinomas (PDAC), and other cancers. Among the most common point mutations in KRAS is G12D, an example of an oncogenic sequence present in tumor cells but not normal cells. We developed peptide nucleic acid (PNA) oligomers that selectively bind KRAS G12D sequences and fused them with novel cell-penetrating peptide flanking regions (CPP-PNA-G12D) then evaluated them. Electrophoretic mobility shift assays demonstrated in vitro binding to and selectivity for KRAS G12D over wild-type KRAS and KRAS G12C. Cells and nuclei were able to uptake CPP-PNA-G12D at high efficiency as shown by fluorescent microscopy and flow cytometry. Cell viability assays showed a striking dose-response effect in on-target cells expressing KRAS G12D, while relatively sparing off-target cells expressing KRAS G12C. CPP-PNA-G12D constructs were effective against a panel of PDAC cell lines and in female Balb/c mice bearing patient-derived xenografts. These results show promise for an enhanced PNA-delivery peptide conjugate strategy as a potential therapeutic strategy to selectively target KRAS mutant cancer cells, with the potential to expand this technology to additional cancer-derived mutant oncogenes.

## 1. Introduction

Conventional chemotherapies non-selectively target growing tumor cells and normal cells causing high toxicities, highlighting the need for more selective and programmable targeted therapeutic strategies. KRAS is part of the RAS family of genes, which includes NRAS and HRAS, and plays a crucial role in driving proliferative cellular signaling pathways. KRAS functions as a small GTPase that toggles between an active and inactive state, driven by the binding of guanosine triphosphate (GTP) or guanosine diphosphate (GDP). KRAS-GTP sends signals that regulate cell growth, proliferation, and survival. In normal cells, KRAS tightly regulates these signals, but mutations can cause KRAS to remain in a constitutively active state, promoting uncontrolled cell proliferation and cancer. KRAS mutations occur in 25–30% of all human cancers, including three of the worst prognosis cancers with approximately 20% of non-small cell lung cancers (NSCLCs), 40% of colorectal cancers (CRCs), and 73–90% of pancreatic ductal adenocarcinoma (PDAC) [1,2,3]. KRAS is most commonly mutated at codon 12 [4]. KRAS inhibitors currently approved by the FDA selectively target the single mutation in NSCLC, CRC, and more recently PDAC. Adagrasib and sotorasib are KRAS G12C inhibitors that rely on irreversible covalent linkage between the inhibitor and the mutant cysteine, making them ineffective against other KRAS mutations [5,6,7]. Experimental tri-complex pan-KRAS inhibitors currently in the clinic target other alleles, as well as wild-type KRAS, NRAS, HRAS, and other structurally related proteins [5,8]. The extent to which pan-KRAS inhibitors’ ability to inhibit wild-type RAS proteins will result in undesirable toxicity in normal cells remains unclear, highlighting the need to develop alternative approaches to mutant KRAS inhibition [1,8].

Peptide nucleic acids (PNA) have nucleotide sequences analogous to DNA, except they have modified peptide backbones instead of ribose-phosphate backbones [9]. PNAs were conceptualized and synthesized as more stable, flexible molecules capable of resisting enzymatic degradation and binding strongly to DNA or RNA targets. Their design aimed to mimic the structure of DNA, but with a N-(2-aminoethyl)-glycine backbone linked to nucleobases instead of the typical ribose-phosphodiester structure of DNA and RNA [10]. PNAs are capable of binding gene sequences many fold more avidly than complementary native DNA. Certain PNAs have been reported to function to repress their targets by hybridizing with a DNA target at orders of magnitude higher affinity than the complementary DNA strand, strand-invading the duplex and blocking transcriptional machinery from transcribing the target sequence [9,11,12]. PNAs may have utility for targeting of mutant genes and molecular diagnostics [13].

Peptide nucleic acids are highly sequence-selective relative to alternatives such as small interfering RNAs, suggesting a therapeutic potential to selectively target diseased cells containing a specific point mutation using a complementary PNA [14]. PNAs have been tested against mutant oncogenes including BRAF, and for suppression of oncogenic miRNAs and lncRNAs in cancer [13,15,16,17]. Previous uses of KRAS G12D PNA employed lipofectamine delivery systems, liposomes, and/or cell-penetrating peptides to enter cells [18,19]. Lipofectamine, a widely used transfection agent, forms complexes with PNAs, enhancing their entry into cells by temporarily destabilizing cell membranes. While effective in in vitro research, lipofectamine has limitations that prevent it from being viable in clinical settings [18].

To achieve successful suppression of mutant KRAS, we employed a variety of strategies to modify PNA conjugates to improve their regional target stability and intracellular and nuclear delivery (Supplementary Figure S1). To optimize PNAs for specificity, we developed peptide nucleic acid oligomers conjugated to nuclear delivery peptides that can target and obstruct transcription, in a sequence- and allele-specific complementary manner against KRAS G12D, a target which is present only to cancer cells. Due to the statistical challenge for the PNA conjugate finding its chromosomal target sequence, stabilizing the PNA oligomer against the chromosomal target may better ensure that the PNA conjugate remains within the chromosomal binding arena and increase the statistical likelihood of binding. The Zimm-Bragg helical model shows that an initial contact of two strands with helical structure forming propensity is the kinetically limiting event for helix formation [20]. As the termini are statistically the most configurationally labile portion of the cell-penetrating peptide/peptide nucleic acid (CPP-PNA) chain, its stabilization against the complementary target increases propensity for helical binding [20]. Stabilizing cationic termini, the most configurationally labile regions, towards the chromosome anionic ribose phosphates offers an effective route to maintaining contact reducing entropic obstacles towards helix formation. Moreover, by better stabilizing these conjugates within the phospholipid bilayer of cellular membranes, the equilibrium of the CPP-PNA conjugate within the cell and nucleus is more quickly achieved as the membrane becomes less of a barrier [16]. We hypothesized that CPP-PNA-G12D constructs would bind KRAS G12D, enter cells, and inhibit the viability and proliferation of cells expressing KRAS G12D using in vitro and in vivo model systems. 

We employed KRAS G12D and non-G12D control cell lines to evaluate the ability of CPP-PNA conjugates targeting KRAS G12D to bind cells, localize to nuclei, selectively impede cell viability of G12D cell lines, and inhibit G12D-driven tumor growth in vivo using patient derived xenografts (PDX). These PNA oligomers differentiated sequences that differed by a single nucleotide base, penetrated cells, and effectively suppressed tumor growth in patient-derived xenografts of KRAS G12D PDAC cells in Balb/c mice. Our findings support the efficacy and the partial specificity of this improved CPP-PNA conjugate strategy.

## 2. Results

### 2.1. PNA Design and Synthesis

The sequence region of each PNA was designed using either a 17- or 20-mer sequence surrounding the region encoding the twelfth amino acid of KRAS G12D (Supplementary Figure S1 and Supplementary Table S1). As is standard in PNA design, base sequences were attached to a repeating peptide backbone, rather than the repeating phosphate backbone found in DNA. The regions surrounding the mutant KRAS sequence were wedged between a series of moieties featuring ionic and cationic regions flanked by hydrophobic regions, designed to improve cell penetration (Supplementary Figure S1 and Supplementary Table S1). All CPP-PNA conjugates were purified then validated by HPLC and matrix-assisted laser desorption/ionization mass spectrometry (MALDI MS) (Supplementary Figure S2).

### 2.2. Effects of G12D-PNA on Binding

**Figure 1:**
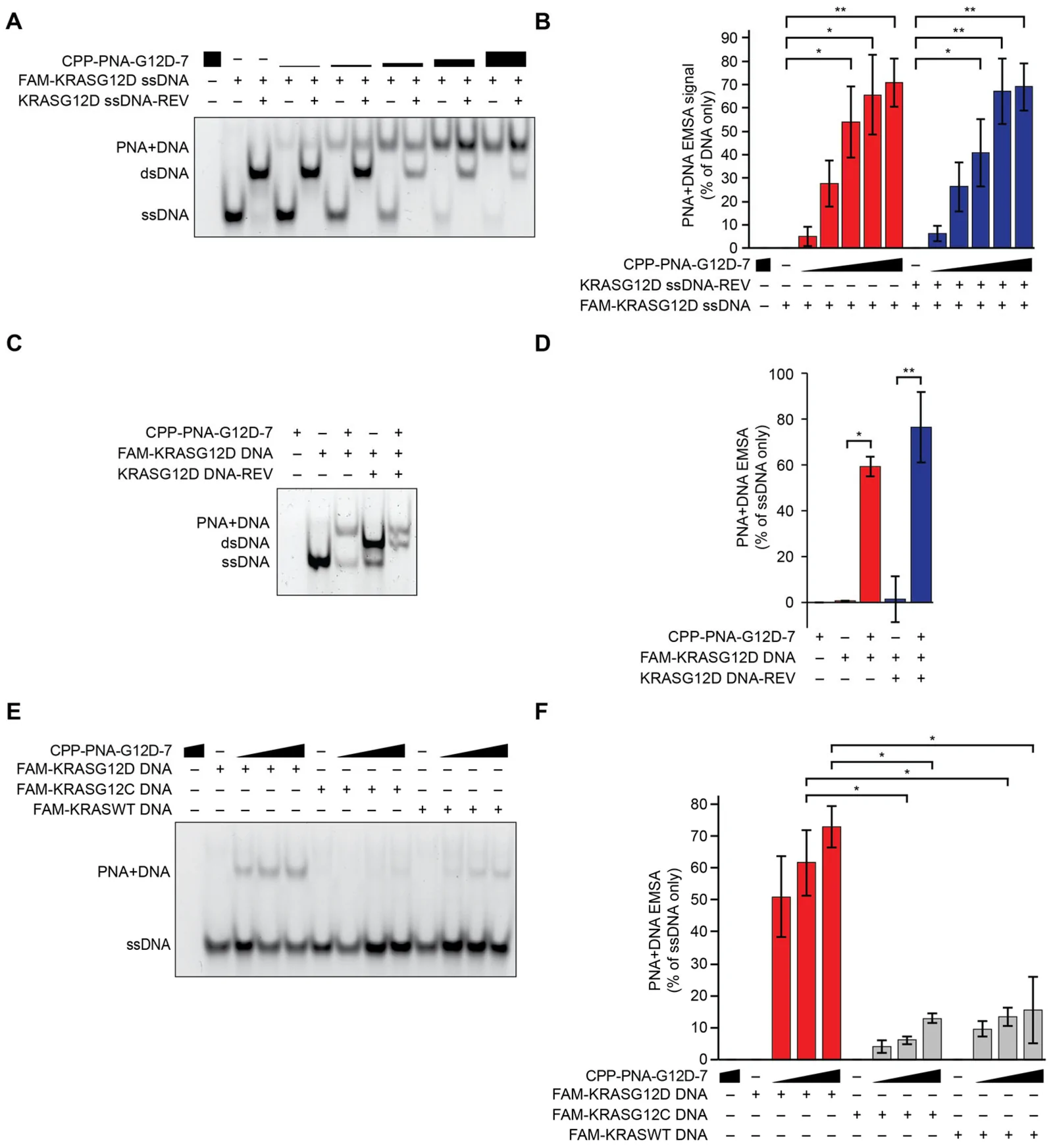
Electrophoretic mobility shift assays (EMSA) and quantification. Plus signs represent the presence of the indicated agent, minus signs in the PNA row represent DMSO vehicle treatment, and minus signs in the subsequent rows represent PBS vehicle treatment. Representative EMSA images shown from n = 3 biological replicates, error bars represent SEM, * *p* < 0.05, ** *p* < 0.01. See Supplementary Table S1 for PNA sequences and Supplementary Table S2 for DNA oligo sequences. Red bars represent CPP-PNA-G12D-7 with complementary KRAS G12D ssDNA, blue bars represent PNA with both forward and reverse KRAS G12D DNA, grey bars represent KRAS G12C ssDNA or KRAS WT ssDNA as indicated, (**A**) CPP-PNA-7, complimentary FAM-labeled KRAS G12D DNA oligo (30 nt), or reverse KRAS G12D oligo (30 nt) at the indicated molar ratios of 1 DNA to 0.25 (lanes 4–5), 0.5 (lanes 6–7), 1 (lanes 8–9), 2 (lanes 10–11), or 4 (lanes 12–13) of CPP-PNA-G12D7, with or without the reverse oligo. (**B**) Quantification of the upper band, representing CPP-PNA-G12D-7 bound to FAM-KRASG12D-ssDNA, as a percentage of signal in the ssDNA only lane. (**C**) EMSA showing ssDNA and complementary ssDNA KRAS G12D sequences pre-annealed prior to incubation with CPP-PNA-G12D-7 in a 1:4 molar ratio. (**D**) Quantification of three experiments in (**C**). (**E**) Comparative EMSA showing the relative binding of FAM-labeled DNA fragments encoding KRAS G12D, KRAS G12C, and KRAS WT at 1:2 (lanes 3, 7, and 11), 1:1 (lanes 4, 8, and 12), and 2:1 (lanes 1, 5, 9, and 13) molar ratios. (**F**) Quantification of three biological replicate experiments in (**E**).

We set out to evaluate the ability of CPP-PNA-G12D to bind KRAS G12D DNA. In vitro electrophoretic mobility shift assay (EMSA) using molar ratios of 1:4, 1:2, 1:1, 2:1, or 4:1 of CPP-PNA-G12D-7 to FAM-labeled complementary single-stranded or double-stranded KRAS G12D DNA oligos demonstrated clear hybridization in a dose-dependent manner (Figure 1A,B, Supplementary Figure S3A). At higher molar ratios, CPP-PNA-G12D-7 outcompeted the complementary DNA sequence to reduce the amount of dsDNA (Figure 1A,B, Supplementary Figure S3A). To evaluate the ability of the PNA to invade dsDNA, we performed EMSA experiments where PNA constructs were introduced to pre-annealed double stranded KRAS G12D DNA. The CPP-PNA-G12D-7 outcompeted the complementary DNA strand to form a binary complex with pre-bound DNA (Figure 1C,D). In EMSA experiments evaluating the selectivity of CPP-PNA-G12D-7 for the KRAS G12D sequence relative to the KRAS WT and KRAS G12C sequences, the PNA was found to be significantly more selective for complementary G12D DNA relative to highly similar G12C DNA or WT DNA sequences (Figure 1E,F, Supplementary Figure S3B).

### 2.3. PNA Localization

**Figure 2:**
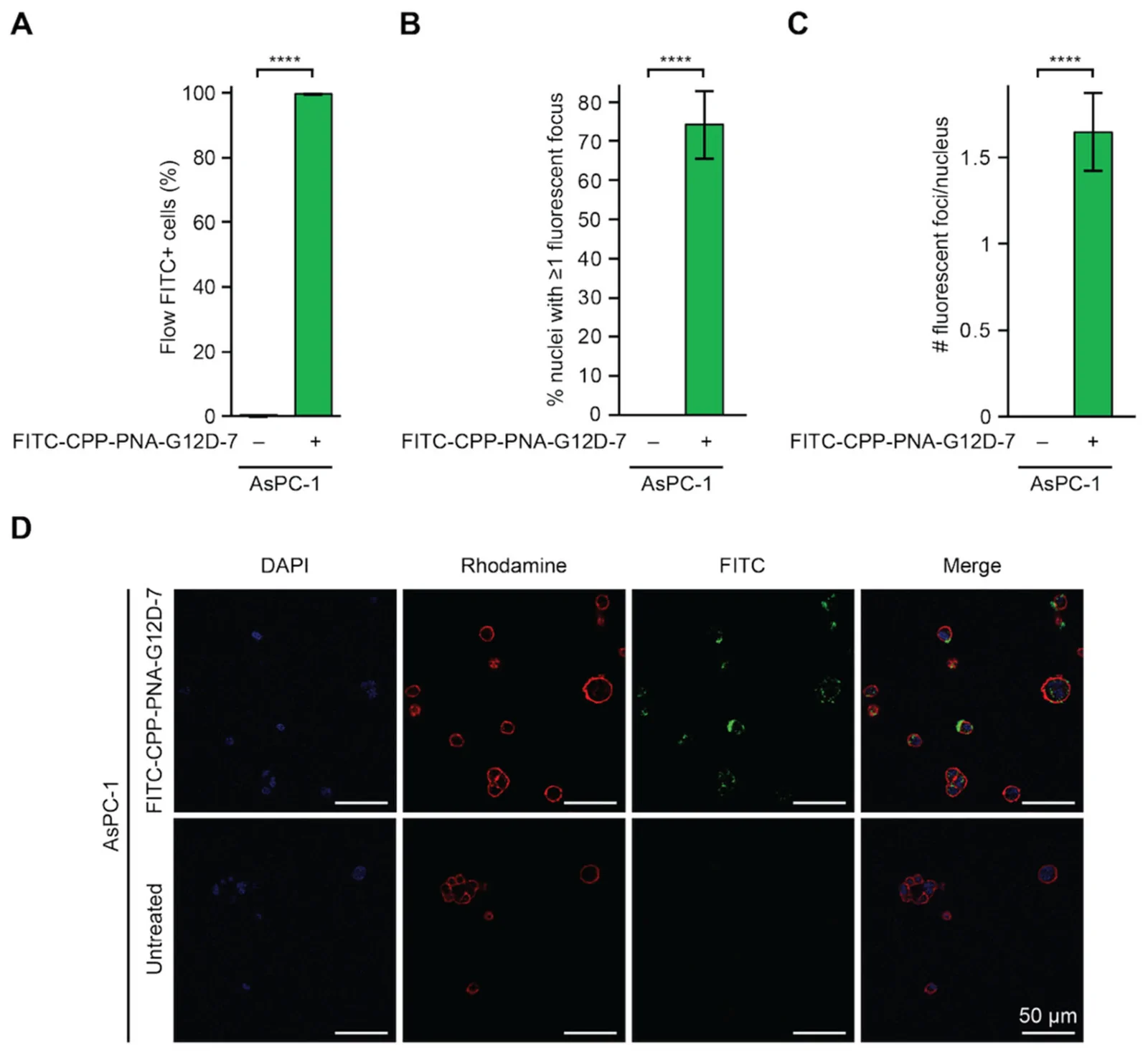
Cell-penetrating peptide PNA conjugates enter cells and nuclei. (**A**) AsPC-1 cells were treated with 500 nM FITC-CPP-PNA-G12D-7 or vehicle for 4 h prior to flow cytometry. Shown are the proportions of FITC+ gated live cells, n = 3 per condition. (**B**,**C**) Image quantification analyses of the percentage of cell nuclei containing at least 1 fluorescent focus (**B**) and the number of distinct fluorescent foci per nucleus (**C**) in AsPC-1 cells treated with 500 nM FITC-CPP-PNA-7 or vehicle for 4 h. (**D**) Representative fluorescent microscopy images of AsPC-1 cells. For (**C**,**D**), AsPC-1 cells were treated with 500 nM FITC-CPP-PNA-G12D-7 or vehicle for 4 h. n = 5 fields per condition. Red represents rhodamine-phalloidin which binds to actin, blue represents DAPI which labels nuclei, green represents FITC-CPP-PNA-G12D-7. Images are uncropped. For all panels, statistical analysis was performed using two-tailed *t*-tests, error bars represent SEM, **** *p* < 0.0001.

To quantify the ability of our modified PNAs to enter cancer cells, we created FITC-CPP-PNA-G12D-7, a FITC-labeled version of CPP-PNA-G12D-7 (Supplementary Figure S1). We tested the ability to bind cells by flow cytometry in three lung and pancreatic cancer cell lines, AsPC-1, H358, and H1975. Cells were treated with 500 nM FITC-CPP-PNA-G12D-7 for 4 h, followed by multiple washes to remove any remaining extracellular PNAs, and assessment for FITC by flow cytometry (Figure 2A and Supplementary Figure S4A–C). DAPI was used to label cell nuclei and rhodamine phalloidin was used to label actin in cytoplasmic regions. In all cell lines, over 99% of cells showed FITC signal, indicating ubiquitous presence of the labeled PNA. 

We further assessed the subcellular localization of FITC-CPP-PNA-G12D-7 in the cytoplasm and nucleus by fluorescent microscopy. AsPC-1 cells dependent upon KRAS G12D and NCI-H358 cells dependent upon KRAS G12C were treated with FITC-CPP-PNA-G12D for 4 h prior to staining for rhodamine-actin and DAPI. Fluorescent puncta frequently localized to the nuclei of both G12D and G12C cell lines (Figure 2B–D and Supplementary Figure S4D–F). Across five fields, approximately 74% of AsPC-1 cell nuclei had at least one fluorescent focus (Figure 2B), with an average of approximately 1.6 foci of FITC signal per nucleus (Figure 2C), consistent with an ability of CPP-PNA-G12D conjugates to readily enter nuclei.

### 2.4. CPP-PNA-G12D Inhibition of Viability of KRAS G12D-Dependent Cells

**Figure 3:**
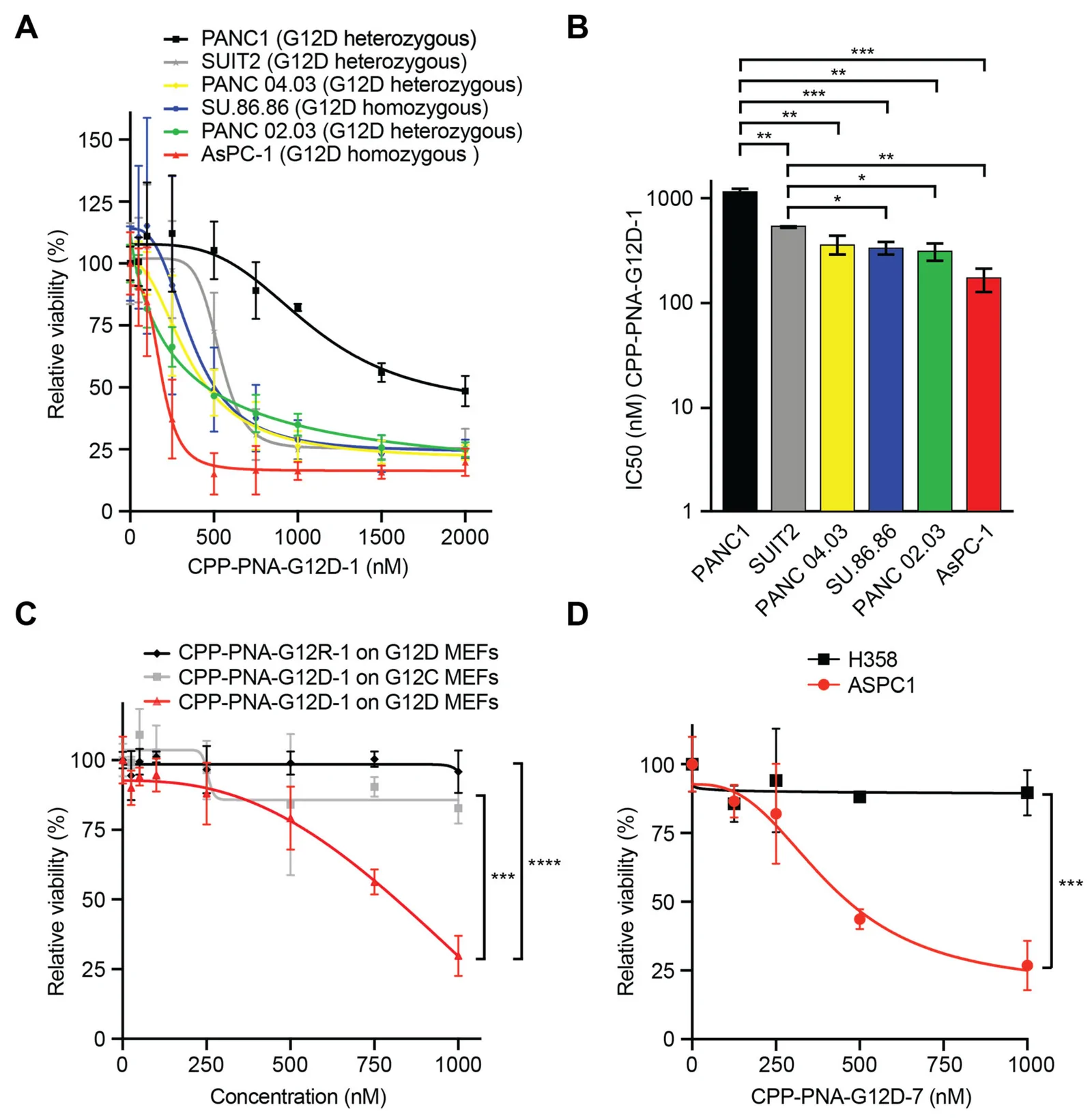
Efficacy and specificity of CPP-PNA-G12D against cell lines. (**A**) Dose-response viability assays for six KRAS G12D PDAC cell lines following 7 days of treatment with media changes every 48 h, assessed by CellTiter-Glo and normalized to vehicle treatment. (**B**) Half-maximal inhibitory concentration (IC50) of CPP-PNA-G12D-1 on six cell lines in nanomolar. (**C**) Rasless MEFs overexpressing cDNAs with KRAS G12D (black and red lines) or KRAS G12C (grey line) following 7 days of treatment with CPP-PNA-G12D-1 or CPP-PNA-G12DR-1, assessed by CellTiter-Glo and normalized to vehicle treatment. (**D**) Relative viability of sensitive AsPC-1 cells, dependent upon KRAS G12D, and resistant H358 cells, dependent upon KRAS G12C, following 7 days of treatment with the indicated doses of CPP-PNA-G12D-7, assessed by CellTiter-Glo and normalized to vehicle treatment. For all experiments, n = 3, error bars represent SEM, and * *p* < 0.05, ** *p* < 0.01, *** *p* < 0.001, **** *p* < 0.0001.

To assess whether other KRAS G12D-dependent cell lines would be sensitive to inhibition with CPP-PNA-G12D-1, we treated a panel of six PDAC lines containing KRAS G12D and a variety of other secondary and tertiary mutations (Figure 3A). In a dose-response experiment ranging from 0–2000 nM of CPP-PNA-G12D-1, the PNA suppressed viability in a dose-dependent manner across the panel of KRAS G12D mutant cell lines (Figure 3A,B). The IC50 values were lowest in the KRAS G12D homozygous line ASPC-1 (170 nM) and were highest in the KRAS G12D heterozygous line PANC1 (1149 nM) (Figure 3B).

Selectively targeting mutant KRAS over wild-type KRAS is important for the ability to target cancer cells while sparing wild-type cells. To evaluate the specificity of our CPP-PNA-G12D conjugates, we employed isogenic MEF cell lines lacking endogenous KRAS, HRAS, and NRAS whose growth was rescued by cDNAs expressing either KRAS G12C or KRAS G12D [21]. In 7-day dose course experiments with drug refreshes every 48 h, the CPP-PNA conjugate targeting G12D displayed a dose-dependent and genotype-selective effect in G12D MEFs (Figure 3C). However, CPP-PNA-G12D-1 had no effect on negative control G12C MEFs, which only contain an off-target KRAS mutation. Congruently, CPP-PNA-G12D-1 displayed strong dose-dependent effects against the KRAS G12D line AsPC-1 cells but minimal effects against the KRAS wild-type cell line H1975 (Supplementary Figure S5). We developed control CPP-PNA conjugates with the same backbone but with a nucleotide sequence targeting G12R called CPP-PNA-G12R-1 (Supplemental Figure S1A). Congruently, CPP-PNA-G12R-1 was incapable of inhibiting MEFs expressing KRAS G12D (Figure 3C). These results, showing that on-target inhibitions are effective in a dose-dependent manner, but mismatching the mutation in either the CPP-PNA or the target cells prevents inhibition, reinforcing the selective nature of this CPP-PNA conjugate design in cancer cells.

To determine whether the effect of CPP-PNA-G12D on cell viability was dependent upon the PNA being unbound by DNA prior to treatment, CPP-PNA-G12D-1 was pre-annealed with a single-stranded complementary DNA oligo to create heteromeric PNA/DNA oligo complexes. ASPC-1 and H358 cells were then treated with a dose course of these complexes (Supplementary Figure S6). As expected, pre-bound PNA/DNA heteromeric oligo complexes were not effective at blocking cell viability in any genotype, including on-target G12D cells, confirming that being single-stranded is required for the mechanism of action (Supplementary Figure S6).

### 2.5. Modifications to Cell-Penetrating Peptide and Length of KRAS Binding Sequence

Using the initial CPP-PNA-G12D-1 design, we iterated upon the sequence and structure toward increasing solubility and engagement with DNA. We employed several strategies to improve on our initial G12D PNA design including motifs to optimize specificity, cell penetration, and solubility. The KRAS G12D homologous base pair (bp) sequences were extended from 17 bp up to 20 bp to increase specificity, creating a genome-unique sequence. Alternative lengths, identities, and orders of the flanking region were designed and synthesized using longer lysine-rich amino acid flanking regions that were interspersed with histidines, arginines, or aspartic acids to make regions more or less cationic (Supplementary Figure S1A and Supplementary Table S1). CPP-PNA conjugates were designed with cell-penetrating regions concentrated in the N-terminal, the C-terminal, or both. 

To evaluate the efficacy and specificity of these additional PNA backbones and longer complementary sequences at selectively targeting cells with KRAS G12D relative to cells with wild-type KRAS, we conducted 7-day relative dose course viability assays (Figure 3D and Supplementary Figure S7). These assays used KRAS G12D selective CPP-PNA conjugates with varying backbone content on either sensitive KRAS G12D AsPC-1 cells or insensitive KRAS G12C H358 cells. Each of these G12D-targeting CPP-PNA conjugates demonstrated significant selectivity for inhibiting growth of cells with KRAS G12D relative to those with KRAS G12C (Figure 3D and Supplementary Figure S7). The most effective and selective version of our KRAS G12D PNA was CPP-PNA-G12D-7 (Figure 3D). This variant contained a 20 nucleotide KRAS G12D homologous region flanked on both ends by ε-palmitoyl-lysines. It encompassed aspartic acid and lysine-rich flanking regions on the 5′ end and a lysine-rich region on the 3′ end (Supplementary Table S1). This candidate demonstrated an improved ability to inhibit cell viability in on-target cells, while sparing off-target cells, with the lowest IC50 in AsPC-1 cells (Supplementary Figure S7F).

### 2.6. Growth Inhibition of KRAS G12D Patient-Derived Xenografts In Vivo

**Figure 4:**
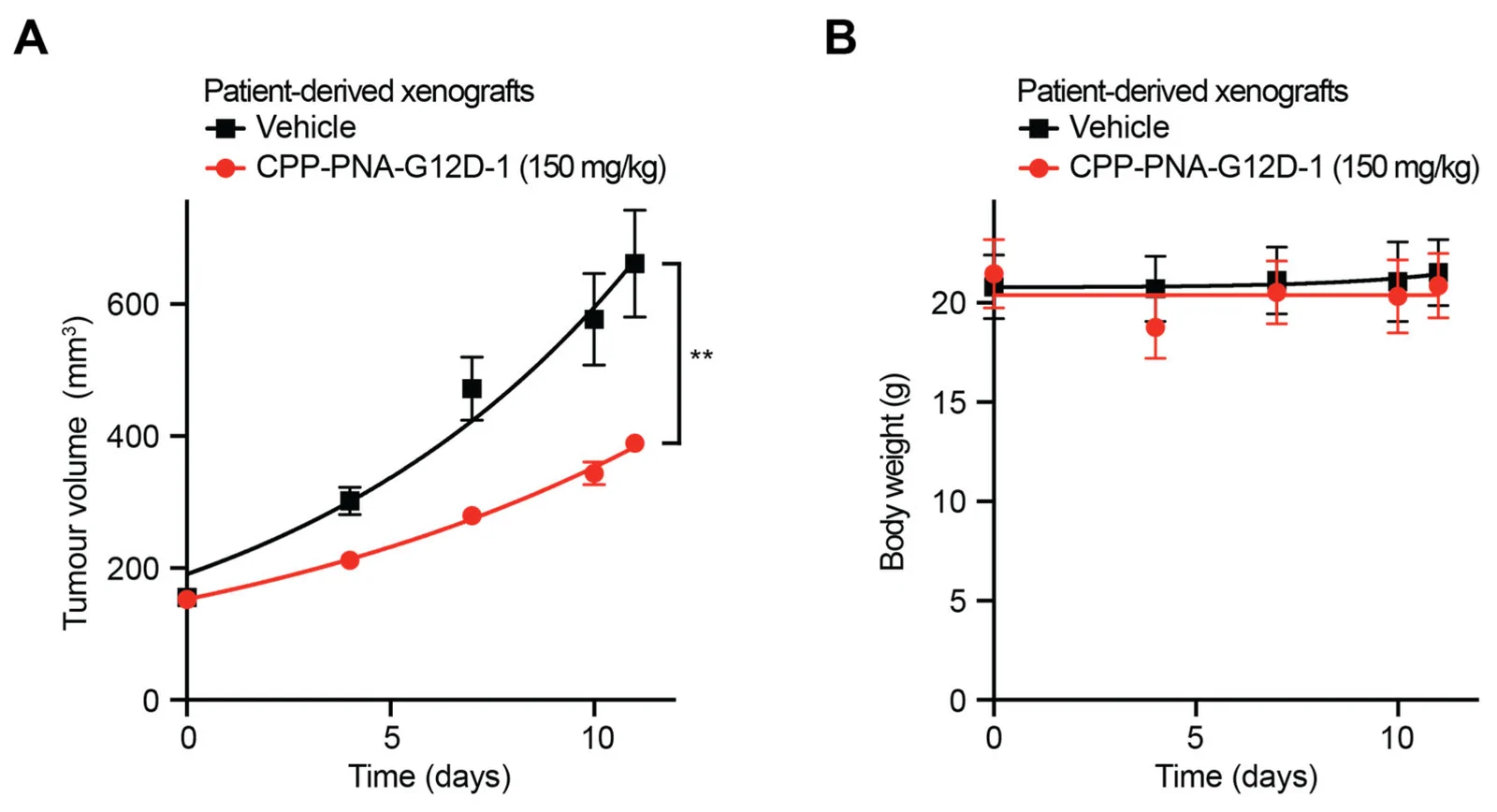
CPP-PNAs are effective and non-toxic in patient-derived xenografts of KRAS G12D PDAC. (**A**,**B**) Tumor volume (**A**), and body weight (**B**), over time for mice bearing patient-derived PDAC xenografts treated with 150 mg/kg of CPP-PNA-G12D-1. Statistical analysis was performed using area under curve followed by *t*-test, n = 3 tumors per condition, error bars represent SEM, ** *p* < 0.001.

To evaluate the efficacy and safety of CPP-PNA-G12D in vivo, a patient-derived xenograft (PDX) from a human PDAC sample with a KRAS G12D mutation was grown in the flanks of Balb/c nude mice. Mice treated with 150 mg/kg by intraperitoneal injection of CPP-PNA-G12D-1 demonstrated significantly less tumor growth relative to vehicle-treated controls (Figure 4A). Upon dissection, precipitate was observed along the abdominal wall, and some hepatic inflammation occurred, implying that only a fraction of the PNA was absorbed. Treated mice did not show a significant change in body weight (Figure 4B). Thus, CPP-PNA-G12D may represent an effective strategy for treating KRAS mutant cancers in vivo. 

## 3. Discussion

In the present study, we extended and rearranged the cationic delivery peptide motif toward improved delivery motifs conjugated to complementary PNAs to suppress an oncogenic target, mutant KRAS [16]. The PNA sequences employed in this study were complementary to the reverse DNA strand rather than the coding DNA strand. This design precludes the possibility that the suppressive mechanism of complementary PNA action is through direct antisense binding to single stranded KRAS messenger RNA. These oligonucleotide analogues lend themselves well to automated peptide synthesis incorporating stabilizing peptides, which make these oligomers considerably more membrane-permeable and more conducive to helical binding. The PNA oligomer differentiated sequences differed by a single nucleotide base, demonstrating partial specificity for KRAS G12D over other KRAS sequences. Due to the statistical challenge for the PNA complement finding its chromosomal target sequence, the cationic termini of the CPP-PNA oligomer are stabilized against the polyanionic chromosomal target, to better ensure that the PNA conjugate remains within the chromosomal binding arena and improve initiation of duplex binding. These results support the efficacy and semi-selectivity of this improved PNA-delivery peptide conjugate strategy. PNAs are relatively resistant to intra- and extracellular enzymes, show single base-pair mismatch discrimination, when conjugated to transport/nuclear-locating peptides are permeable to nuclear and cell membranes, and suppress target genes at nanomolar concentrations in cell culture [16]. The present study focused on KRAS G12D as it is a prevalent cancer-causing mutation. CPP-PNA-G12D designs differed from earlier KRAS PNA designs in several significant ways. Previous KRAS targeting PNA applications utilized lipofectamine or 2D-layered Mg-Al layered double hydroxides (LDHs) as carriers for delivery, which may be a limitation in clinical settings [18,19]. Due to the ionic and cationic modifications added to the ends of the PNAs used in this study, they were capable of penetrating both the cell and nuclear membranes and localizing within the nucleus of KRAS mutant cells. Stereochemical enantiomers of lysine, histidine, aspartic acid, and arginine were employed in different combinations alongside flanking acetylation and palmitoylation groups. Prior PNAs were designed to target the KRAS mRNA through complementarity with the forward coding sequence, whereas our approach focuses on targeting the mutation exclusively at the DNA level through complementarity with the reverse strand rather than the coding strand [18,19]. The CPPs in this study were effective at lower concentrations than PNA conjugated to traditional nuclear localization signals [16]. When PNA was pre-bound to DNA prior to treatment, it demonstrated no efficacy against on-target cells, which may confirm that free nucleotides are important to penetrate the phospholipid anionic membranes.

PNA technologies have multiple potential applications in biology. KRAS PNAs have been previously used as reagents for peptide nucleic acid-locked nucleic acid polymerase chain reaction (PNA-LNA PCR) to detect KRAS mutations in surgically resected lung cancer samples, with more efficient detection than traditional PCR methods [22]. Available cancer chemotherapeutics often do not definitively distinguish between normal cells and cancer cells, as they target pathways and signaling mechanisms found and used in both [23,24,25,26,27]. Using PNAs to reversibly obstruct the transcription or replication of a gene sequence, such as an oncogene, that is both crucial for tumorigenesis and specific to the cancer cell itself, offers a potential route to facilitate targeting cancer cells specifically through their genetic distinctiveness. Cell lines with homozygous KRAS G12D tended to be more sensitive than those that are heterozygous, possibly because wild-type KRAS can provide a bulwark against inhibition of mutant KRAS (Figure 3A,B) [28,29,30]. In PDX models, tumor sizes were reduced without weight loss. The observation of acute precipitation at the injection site suggests that in vivo solubility remains a challenge, and that improved solubility for in vivo systems would be expected to increase efficacy in mice. Further studies on solubility, formulation, and systemic tolerability will be important for optimization, as improved solubility is likely to correlate with improved tumor responses. The sample size of the PDX experiments was limited; therefore, validation of these reagents in larger cohorts and across additional PDX models represents an important direction for future studies. These results indicate that PNAs show promise as both a tool for studying point-mutation-driven tumors and as a possible therapeutic strategy capable of sparing normal cells while selectively targeting mutant cancer cells. Our findings that CPP-PNA-G12D inhibited on-target KRAS G12D pancreatic ductal adenocarcinoma cell lines and patient-derived xenografts provide proof-of-principle for the efficacy and selectivity of the programmable and modular cell-penetrating peptide conjugated PNA technology that may have applications against many point-mutated oncogenes driving cancer.

## 4. Materials and Methods

### 4.1. Cell Lines

Cell lines were obtained from the American Type Culture Collection (ATCC, Manassas, VA, USA). All cell line identities were genetically confirmed using short tandem repeat analysis (ATCC, Manassas, VA, USA #135-XV). All cell lines tested negative for mycoplasma using MycoAlert^®^ Mycoplasma Detection Kit (Lonza, Washington, DC, USA #LT07-318). The growth conditions for each cell lines were: AsPC-1 (RRID:CVCL_0152), NCI-H1975 (RRID:CVCL_1511), NCI-H358 (RRID:CVCL_1559), SU.86.86 (RRID:CVCL_3881), SUIT-2 (RRID:CVCL_3172) cells were maintained in RPMI-1640 (Gibco, Grand Island, NY, USA #11875119), 10% fetal bovine serum (FBS) (R&D Systems, Minneapolis, MN, USA #S11550H), and 1% Penicillin/Streptomycin/L-Glutamine (PSG) (Gibco, Grand Island, NY, USA #10378016) as their complete media. PANC.02.03 (RRID:CVCL_1633) and PANC.04.03 (RRID:CVCL_1636) cells were maintained in RPMI-1640 (Gibco, Grand Island, NY, USA #11875119), 10% FBS, and 1% PSG, with the addition of 20 U/mL human recombinant insulin (Sigma-Aldrich, Saint Louis, MO, USA #I-1882) to create their complete media. PANC-1 (RRID:CVCL_0480) cells were maintained in DMEM (Gibco, Grand Island, NY, USA #11965092), 10% FBS, and 1% PSG as their complete media. Rasless MEFs expressing KRAS G12C or KRAS G12D (NCI RAS Initiative/Leidos Biomedical Sciences, Frederick, MD, USA) were cultured and maintained in DMEM (Gibco, Grand Island, NY, USA #11965092), 10% FBS, and 1% PSG, with the addition of 4 µg/L Blasticidin S (Thermo Scientific Chemicals, Waltham, MA, USA #J61883FPL) to create their complete media. All cells were maintained at 5% CO_2_ in a 37 °C humidified incubator. Live cells used for assays were counted using 1:1 Trypan Blue (Invitrogen, Carlsbad, CA, USA #T10282) and a Countess 3 Automated Cell Counter (Invitrogen, Carlsbad, CA, USA #AMQAX2000) prior to plating. 

### 4.2. PNA Synthesis and Validation

PNAs were designed in collaboration with Oncogenuity, Inc. (Bay Harbor, FL, USA). PNA constructs have 17 to 20 base pairs of the KRAS nucleotide sequence and each PNA includes the nucleotides encoding the aspartic acid at position 12 near the center. See Table S1 for full sequences. PNAs were synthesized and quality control HPLC and MALDI-MS were performed by PNA Bio (Thousand Oaks, CA, USA). Modifications to PNA oligomers include acetylation (Ac), palmitoylation (palm), or FITC conjugation and are detailed in full in Table S1. High-performance liquid chromatography mass spectrometry (LC-MS) was performed using an Agilent 1100 Series from Agilent Technologies (Santa Clara, CA, USA). Matrix-assisted laser desorption/ionization mass spectrometry was performed using an AXIMA-Assurance from Shimadzu Biotech (Columbia, MD, USA). Quality control was performed by PNA Bio.

### 4.3. Electrophoretic Mobility Shift Assay (EMSA)

Lyophilized KRAS WT, KRAS G12C, and KRAS G12D DNA oligomers were synthesized by Sigma-Aldrich (Saint Louis, MO, USA). Forward sequences were fluorescein-labelled (FAM) and their corresponding reverse complement sequences were not FAM-labelled (Table S2). The DNA sequences used for the EMSA were: FAM-KRASG12D-DNA: [6FAM]-TTGCCTACGCCATCAGCTCCAACTACCACA, FAM-KRASWT-DNA: [6FAM]-TTGCCTACGCCACCAGCTCCAACTACCACA, FAM-KRASG12C-DNA: [6FAM]-TTG CCTACGCCACAAGCTCCAACTACCACA, and REV-KRASG12D-DNA: TGTGGTAGTTGGAGCTGATGGCGTAGGCAA. DNA oligomers were reconstituted in 1× PBS. 0.0683 nmol of the indicated DNA oligo, with or without the indicated molar ratio of PNA, were heated to 90 °C, then gradually cooled to anneal as described in Thadke et al. [31]. Each sample was loaded onto a 10% non-denaturing PAGE in 1× TBE buffer (Thermo Scientific, Waltham, MA, USA #B52) and then separated at 120 V for 60 min. Gel images were processed and visualized by the ChemiDoc (Bio-Rad, Hercules, CA, USA #12003154) via UV-Transilluminator. Uncropped EMSA images are available in the Supplementary Materials (Supplemental Figure S8).

### 4.4. Flow Cytometry

Approximately 50,000 AsPC-1, NCI-H1975, and NCI-H358 cells were incubated with either FITC-CPP-PNA-G12D-7 or negative control DMSO for 4 h. After the incubation, cells were trypsinized, neutralized with normal media, centrifuged at 300× *g* for 5 min at 4 °C, aspirated, resuspended in flow media, and added to a 96-well U-bottom plate (Sigma-Aldrich, Saint Louis, MO, USA #CLS4515). Flow cytometry was performed on a CytoFLEX S flow cytometer (Beckman Coulter, Brea, CA, USA #C09762). Live cells were gated with side-scatter and forward-scatter (Figure 2A and Supplementary Figure S4A,B). FITC-CPP-PNA-G12D-7 cells were gated using parameters in which unstained controls had ≤0.2% positive cells using Flowing Software 2.5.1 (Turku Centre for Biotechnology, Turku, Finland).

### 4.5. Fluorescent Microscopy

Approximately 50,000 AsPC-1 and NCI-H358 live cells were plated on 12-well glass bottom plates (Mattek Corp., Ashland, MA, USA #P12G-1.5-10F). On the following day, cells were treated with either DMSO or 500 nM FITC-labeled PNA (FITC-CPP-PNA-G12D-7) for 4 h. The cells were fixed using 4% formaldehyde (Thermo Scientific Chemicals, Waltham, MA, USA #J60401-AP). After 15 min at room temperature, the formaldehyde was removed and the wells were washed with PBS. The cells were permeabilized using 0.1% Triton X-100 in PBS (Fisher Chemicals, Waltham, MA, USA #BP151-500). After 10 min at room temperature, the wells were washed with PBS. Rockland blocking buffer (Rockland Immunochemicals, Limerick, PA, USA #MB070) was added for 30 min. Wells were washed with PBS. Invitrogen ActinRed™ 555 ReadyProbes™ Reagent Rhodamine phalloidin (Invitrogen #R37112) was mixed with 1% BSA + PBST (Fisher BioReagents, Waltham, MA, USA #BP9703100) and placed in the wells to stain for actin. The plates were incubated on an orbital shaker overnight away from light. The next day, cells were washed with PBS, then mounting media containing DAPI (Vector Laboratories, Newark, CA, USA #H120010) was added to stain for DNA. For imaging, a Nikon A1 Confocal Microscope with NIS-Elements software (version 4.6) was used (Nikon, Melville, NY, USA). Five image fields of each well were captured with 60× objective oil immersion. CellProfiler (Broad Institute, Cambridge, MA, USA) was used to quantify nuclei, dots representing PNAs, and actin, and the same settings were applied to all images, n = 5 per condition.

### 4.6. Viability Assays

For dose-course experiments, live cells were plated at 5000 cells/well in sterile optical-bottom 96-well plates (Thermo Scientific #165306) in their respective complete media on day 1. To reduce the impact of edge effects, triplicates were spatially separated on the plate and edge wells were not read. On day 2, each PNA was diluted in media to desired concentrations, then cells were treated with the indicated concentration of media with PNA or vehicle, with all wells receiving the same dose of vehicle. AsPC-1, NCI-H358 cells were treated for 72 h with no drug refresh or for 7 days with drug refreshes every 48 h. At the experimental endpoint, brightfield microscopy images were taken with EVOS FL Auto Imaging system (Applied Biosystems, Waltham, MA, USA #AMEX-1200). Relative viability was then assessed using CellTiter-Glo^®^ Luminescent Cell Viability Assay (Promega, Madison, WI, USA #G7572) per manufacturer protocol and placed in Varioskan Lux (Thermo Scientific #VLBLATGD2) or a Spectramax i3 (VWR, Radnor, PA, USA #10192-220). Data were normalized to vehicle-treated cells within cell lines.

### 4.7. Mouse Xenografts

Female BALB/c nude mice, an immunodeficient mouse strain commonly used in PDX experiments, of 6–7 weeks of age were injected subcutaneously with patient-derived xenografts (PDX) from pancreatic ductal adenocarcinomas (PDAC) sample PA1301. Mice were purchased from GemPharmatech Co., Ltd. (Nanjing, China). Tumor size was monitored on the indicated timepoints by calipers measurements, and tumor volumes were calculated using the standard formula of V = 0.5 × L × W^2^. Upon reaching approximately 150 mm^3^, mice were randomized into treatment and control groups and tumors were treated on alternating days by intraperitoneal injection with 150 mg/kg of CPP-PNA-G12D-1 (n = 3) or vehicle (n = 3) at 7.1 µL/g on three days per week for two weeks. Mice were continuously monitored for changes in body weight and other standard measures of toxicity or distress. Criteria for exclusion in the study were based on a priori criteria. The order of animals used for measurement and treatment was randomized, and measurements were blinded. Mice were sacrificed on day 11, which represented the pre-established humane endpoints that included nearly reaching a tumor volume of 1000 mm^3^. Upon sacrifice, tumors and other organs were collected and analyzed by gross necropsy. Animal studies were performed in accordance with National Institutes of Health standards and were approved by the Committee on the Ethics of Animal Experiments of Crown Bioscience, Inc. (San Diego, CA, USA). Data was analyzed using area under the curve calculations for the primary outcome measure of tumor size followed by analysis of variance (ANOVA), each performed in GraphPad Prism 10 (RRID:SCR_002798). Graphs were generated in GraphPad Prism 10, *p*-values (* *p* < 0.01), error bars represent standard error of the mean.

### 4.8. Statistical Analyses

Statistical tests are described in figure legends. All analyses were performed in GraphPad Prism 10 (RRID:SCR_002798) or Microsoft Excel (RRID:SCR_016137). For dose-course experiments, data were processed using the area under the curve (AUC); following the processing, *p*-values were calculated using one-way analysis of variance (ANOVA) followed by Tukey’s multiple comparisons test in GraphPad Prism. Mouse xenograft data were processed using the area under the curve (AUC); following the processing, *p*-values were calculated using an unpaired *t*-test in GraphPad Prism. Gated cell proportions and fluorescence data were compared using two-tailed *t*-tests in GraphPad Prism. From the above statistical analyses, *p*-values are shown as: ns represents not significant, * *p* < 0.05, ** *p* < 0.01, *** *p* < 0.001, and **** *p* < 0.0001.

## Data Availability

All data are available in the main text or the Supplementary Materials.

## Supplementary Materials

The following supporting information can be downloaded at: https://www.mdpi.com/article/10.3390/ijms27167158/s1.
