# Supplementary material for: Targeting Oncogenic KRAS Using Peptide Nucleic Acid Oligomers Attached to Cell-Penetrating Peptides
Source: Int J Mol Sci. 2026 Aug 10;27(16):7158. doi: 10.3390/ijms27167158 (PMC13513279; doi:10.3390/ijms27167158)
Supplement: Supplementary file 1 [file ijms-27-07158-s001.zip › Supplementary Materials.pdf]

Supplementary Materials associated with:

Targeting oncogenic KRAS using peptide nucleic acid oligomers attached to cell-penetrating peptides

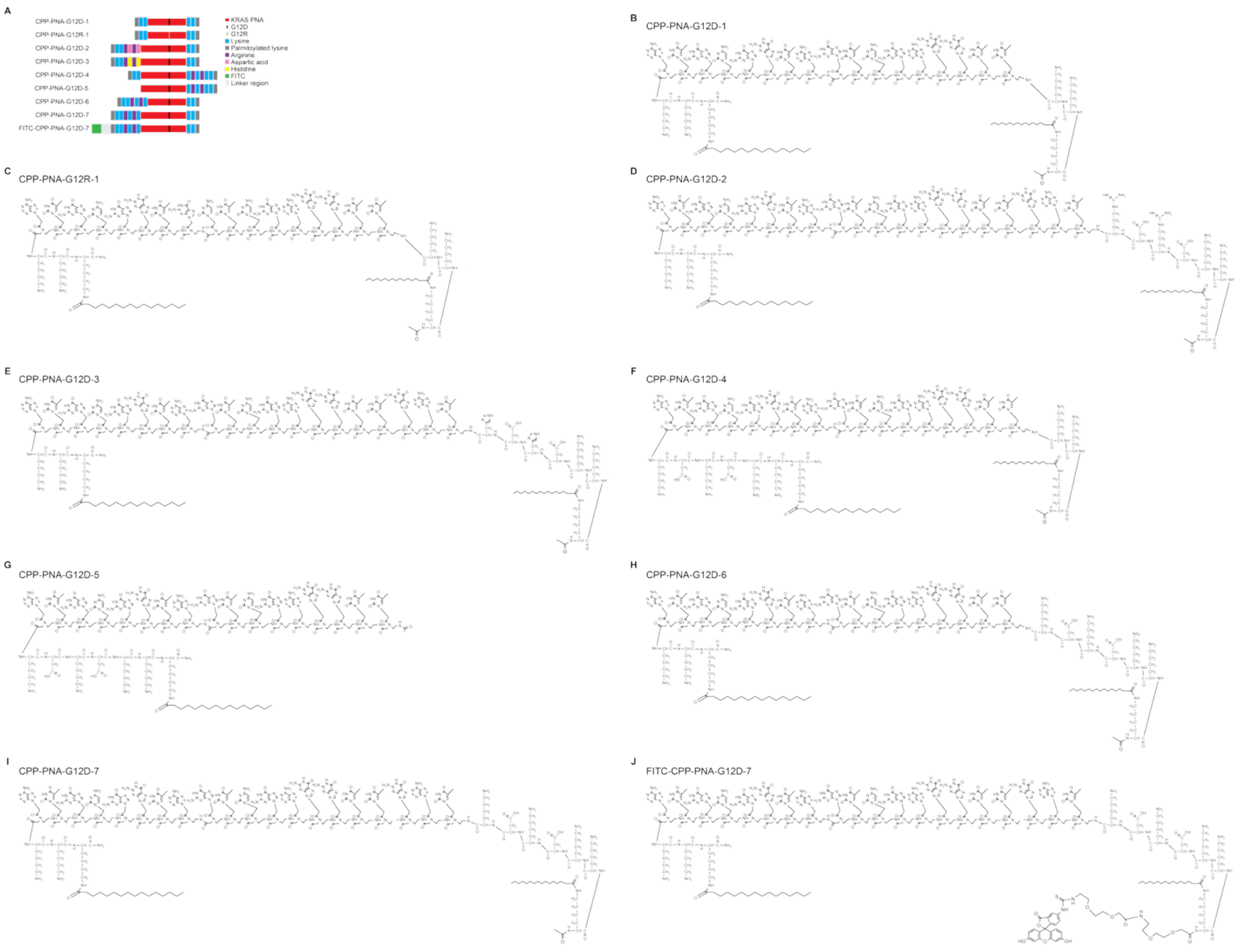

Supplementary Figure S1. PNA structures. **A,** Diagram of PNA structures. Red= complementary KRAS PNA sequence, black = G12D, light brown = G12R, blue = lysine, dark grey = palmitoylated lysine, purple = aspartic acid, pink = arginine, yellow = histidine, orange = acetylation, green = fluorescein isothiocyanate (FITC), light grey = linker region. **B,** ChemDraw structure of CPP-PNA-G12D-1. **C,** ChemDraw structure of CPP-PNA-G12R-1. **D,** ChemDraw structure of CPP-PNA-G12D-2. **E,** ChemDraw structure of CPP-PNA-G12D-3. **F,** ChemDraw structure of CPP-PNA-G12D-4. **G,** ChemDraw structure of CPP-PNA-G12D-5. **H,** ChemDraw structure of CPP-PNA-G12D-6. **I,** ChemDraw structure of CPP-PNA-G12D-7. **J,** ChemDraw structure of FITC-CPP-PNA-G12D-7.

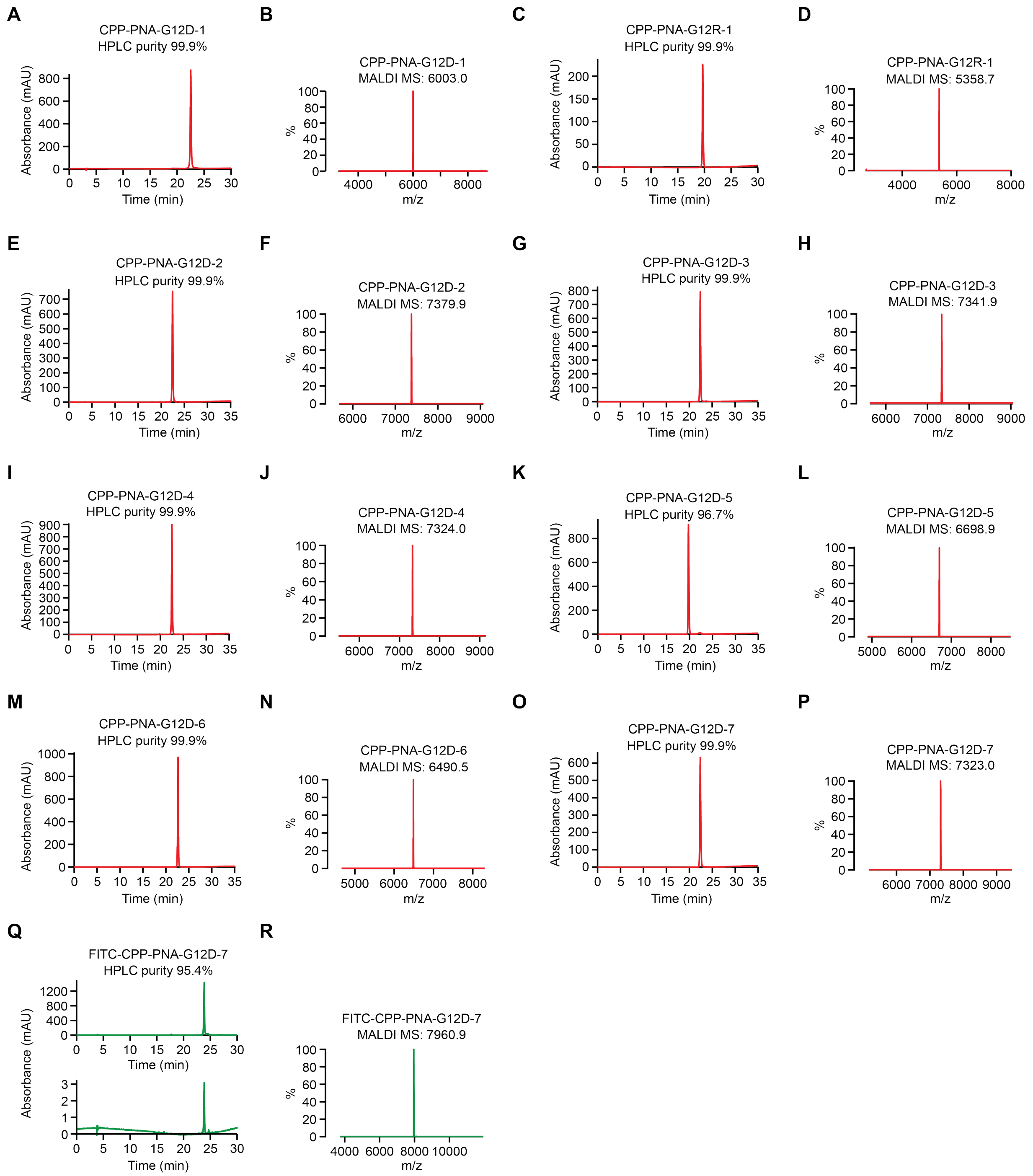

Supplementary Figure S2. Validation of CPP-PNA conjugates. **A-R,** Purity of each CPP-PNA was assessed by high-performance liquid chromatography (HPLC) and matrix-assisted laser desorption/ionization mass spectrometry (MALDI MS). Shown are CPP-PNA-G12D-1 (A-B), CPP-PNA-G12R-1 (C-D), CPP-PNA-G12D-2 (E-F), CPP-PNA-G12D-3 (G-H), CPP-PNA-G12D-4 (I-J), CPP-PNA-G12D-5 (K-L), CPP-PNA-G12D-6 (M-N), CPP-PNA-G12D-7 (O-P), and FITC-CPP-PNA-G12D-7 (Q-R). For MALDI-MS, the indicated peak values represent MS found (M+1).

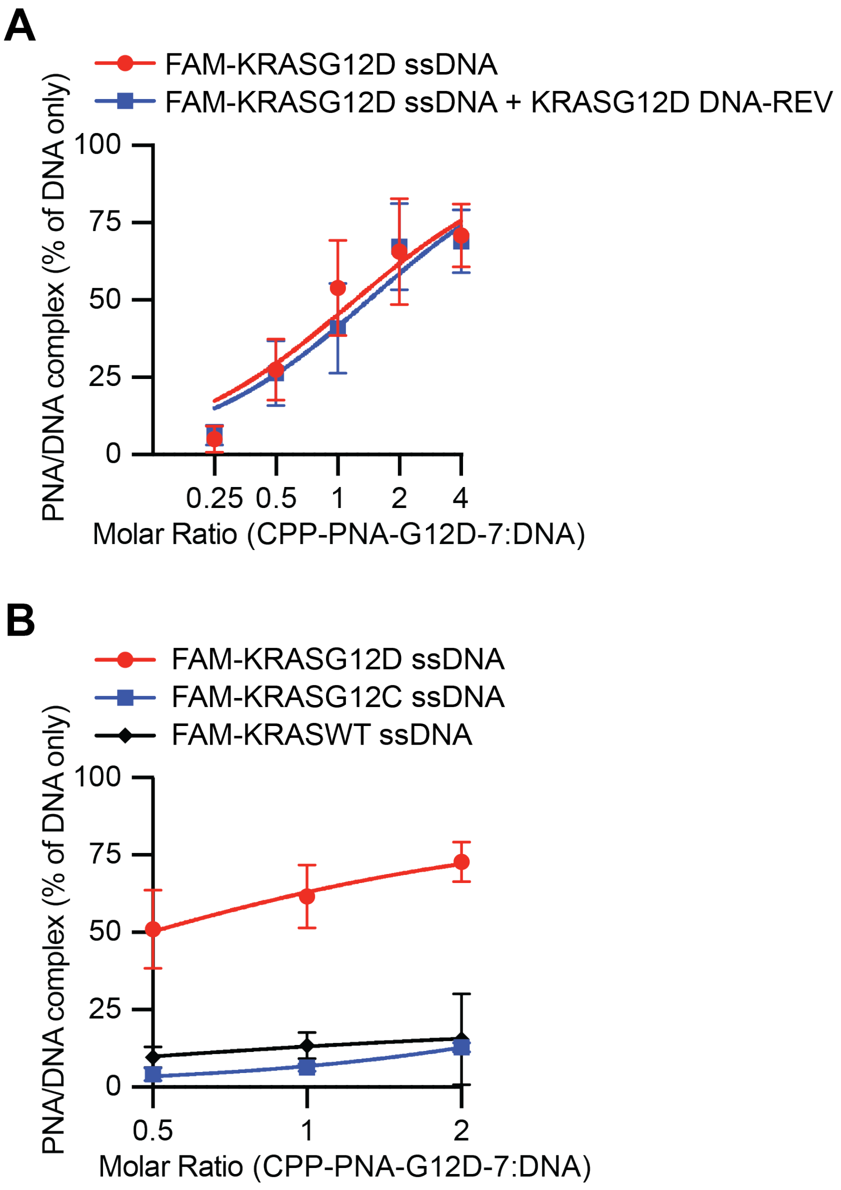

Supplementary Figure S3. Relative amount of PNA/DNA complex from EMSA quantifications, related to Figure 1. **A,** Signal representing complexes were quantified and normalized to the lane containing FAM-KRASG12D ssDNA alone. The K_d of CPP-PNA-G12D-7:FAM-KRASG12D ssDNA was a molar ratio of 1.15:1 and the K_d of CPP-PNA-G12D-7:dsDNA (FAM-KRASG12D + FAM-KRASG12D-REV) was a molar ratio of 1.43:1. **B,** Signal representing complexes were quantified and normalized to the lane containing FAM-KRASG12D ssDNA alone. The K_d of CPP-PNA-G12D-7:FAM-KRASG12D ssDNA was a molar ratio of 0.34:1. The K_d of CPP-PNA-G12D-7:FAM-KRASG12C was a molar ratio of 16.1:1. The K_d of CPP-PNA-G12D-7:FAM-KRASWT was a molar ratio of 0.48:1.

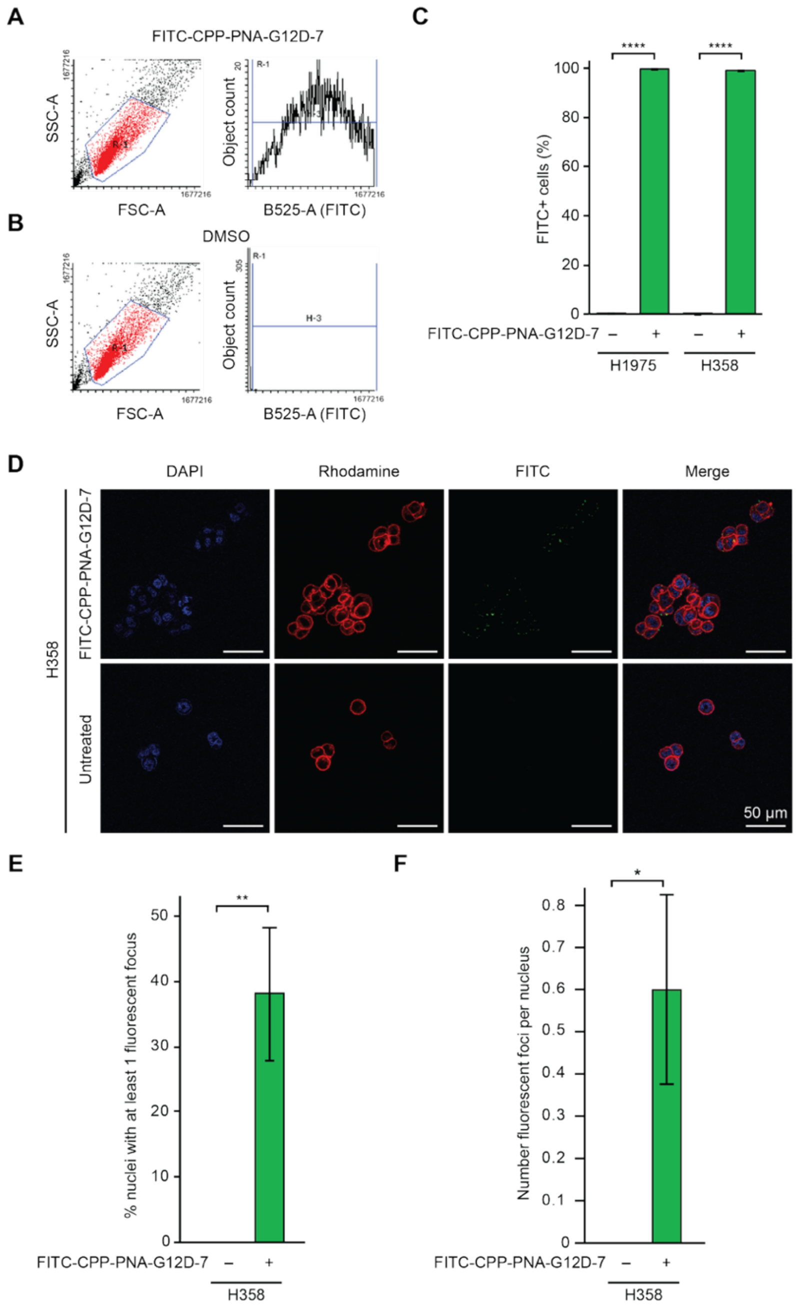

Supplementary Figure S4. Cell penetration of PNAs in additional cell lines. (**A-B**) Representative flow cytometry gating and results for AsPC-1 cells treated with 500 nM FITC-CPP-PNA-G12D-7 (A) or DMSO (B) for 4 hours prior to flow cytometry; n=3, representative images are shown. **C** H1975 and H358 cells were treated with 500 nM FITC-CPP-PNA-G12D-7 for 4 hours prior to flow cytometry. Shown are the proportions of FITC+ gated cells, n = 3 per condition. (**D-F**) Representative fluorescent images (D), and image quantification analyses (E-F), of H358 cells treated with 500 nM FITC-CPP-PNA-G12D-7 for 4 hours. Red represents rhodamine-phalloidin which binds to actin, blue represents DAPI which labels nuclei, green represents FITC-CPP-PNA-G12D-7. n = 5 per condition. For all panels, statistical analysis was performed using two-tailed *t*-tests, * $p < 0.05$, ** $p < 0.01$.

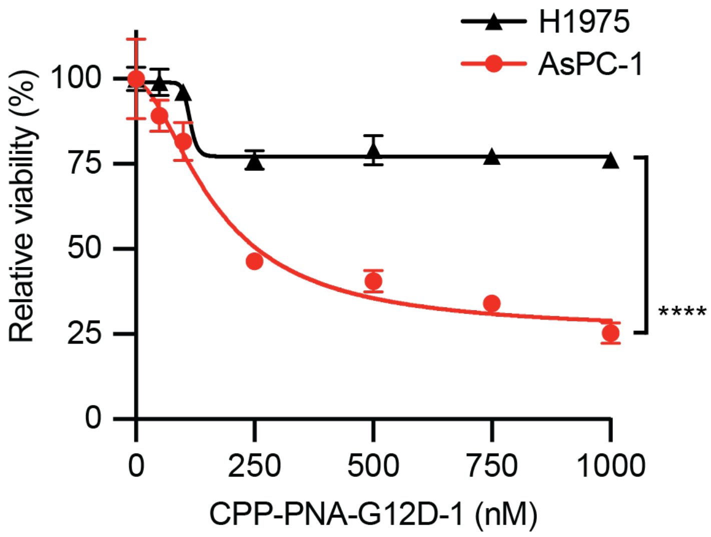

Supplementary Figure S5. Dose-response relative viability by CellTtier-Glo of ASPC-1 or H1975 cells treated with the indicated doses of CPP-PNA-G12D-1 for 72 hours. n=3, error bars represent SEM, **** $p < 0.0001$.

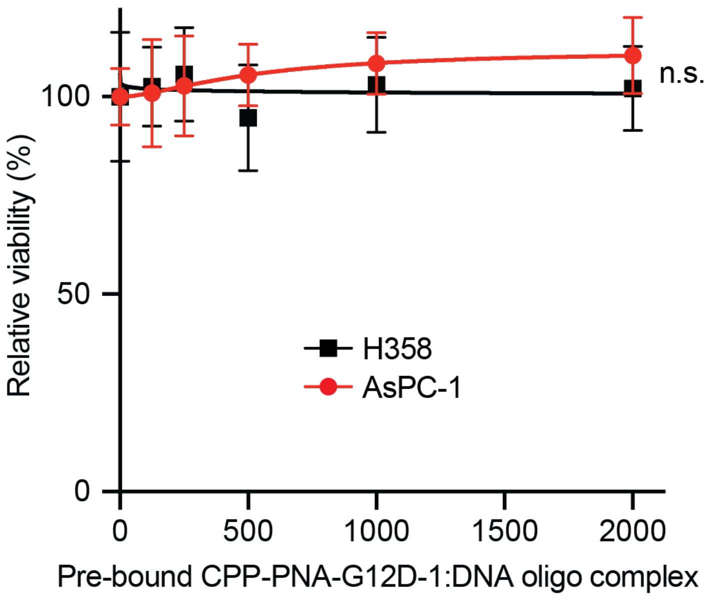

Supplementary Figure S6. Dose-response of relative viability by CellTiter-Glo. Pre-incubation of CPP-PNA-G12D-1 with complementary DNA strands prevented inhibition of AsPC-1 cells (red) or H358 cells (black) at all tested doses in a 7-day dose course with media changes every 48 hours; n = 3, error bars represent SEM, n.s. not statistically significant.

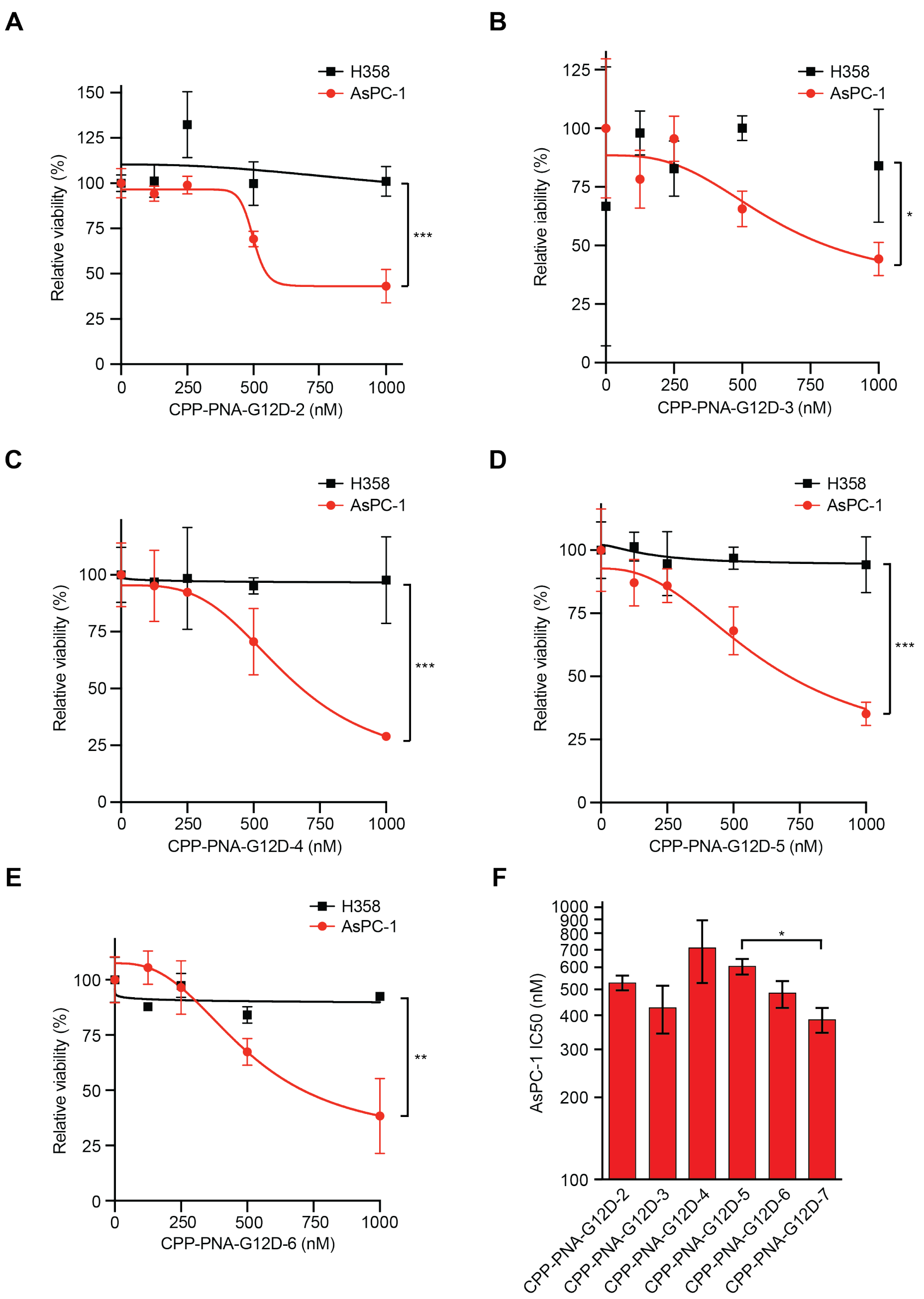

Supplementary Figure S7. PNAs selectively target KRAS G12D mutants. (**A-E**), Relative viability of sensitive AsPC-1 cells by CellTiter-Glo, dependent upon KRAS G12D, and resistant H358 cells, dependent upon KRAS G12C, after 7 days of treatment, with refreshes every 48 hours, with the indicated doses of CPP-PNA-G12D-2 (A), CPP-PNA-G12D-3 (B), CPP-PNA-G12D-4 (C), CPP-PNA-G12D-5 (D), or CPP-PNA-G12D-6 (E). See Supplementary Fig. 1 and Supplementary Table 1 for more information on these CPP-PNA conjugates. Statistical analysis was performed using area under the curve (AUC), n = 3, error bars represent SEM, * $p < 0.01$, ** $p < 0.01$, *** $p < 0.001$. (**F**) Half-maximal inhibitory concentration (IC50) values in nanomolar for AsPC-1 cells treated with the indicated CPP-PNA-G12D conjugates for 7 days with refreshes every 48 hours; n = 3, error bars represent SEM, * $p < 0.01$.

Uncropped EMSA image from Figure 1A

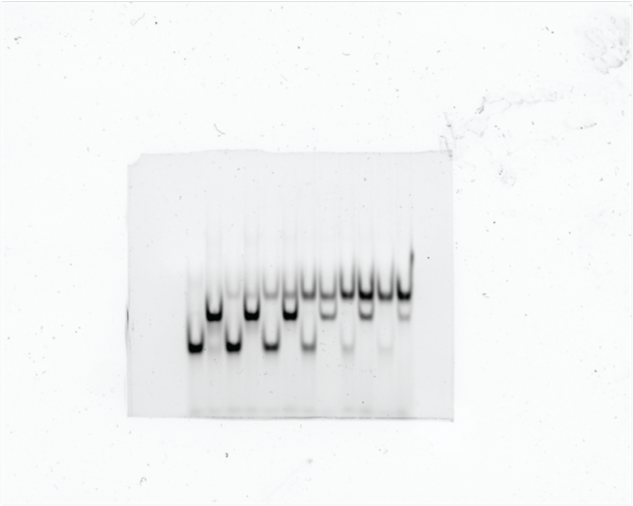

Uncropped EMSA image from Figure 1C

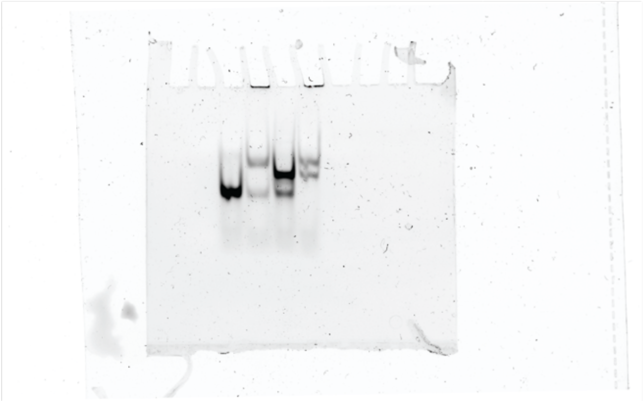

Uncropped EMSA image from Figure 1E

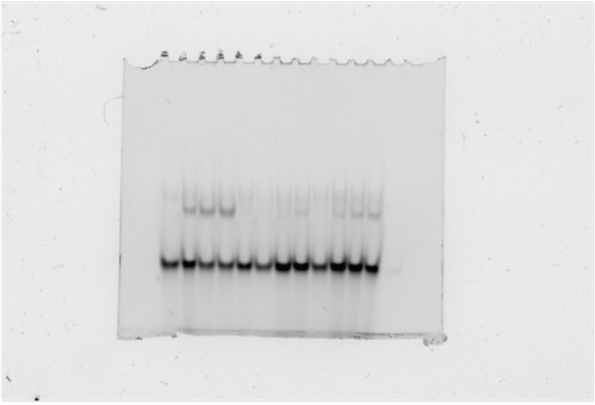

Supplementary Figure S8. Uncropped EMSA images corresponding to Figure 1A, Figure 1C, and Figure 1E.
